# Entropy Measures as Descriptors to Identify Apneas in Rheoencephalographic Signals

**DOI:** 10.3390/e21060605

**Published:** 2019-06-18

**Authors:** Carmen González, Erik Jensen, Pedro Gambús, Montserrat Vallverdú

**Affiliations:** 1 Biomedical Engineering Research Centre, Universitat Politècnica de Catalunya, CIBER of Bioengineering, Biomaterials and Nanomedicine (CIBER-BBN), 08028 Barcelona, Spain; 2 Quantium Medical, Research and Development Department, 08302 Mataró, Spain; 3 Systems Pharmacology Effect Control & Modeling (SPEC-M) Research Group, Department of Anesthesia, Hospital CLINIC de Barcelona, 08036 Barcelona, Spain; 4 Department of Anesthesia and Perioperative Care, University of California San Francisco (UCSF), San Francisco, CA 94143, USA

**Keywords:** cerebral blood flow, rheoencephalography, apnea detection, complexity, approximate entropy (ApEn), sample entropy (SampEn), fuzzy entropy (FuzzyEn), corrected conditional entropy (CCE), Shannon entropy (SE)

## Abstract

Rheoencephalography (REG) is a simple and inexpensive technique that intends to monitor cerebral blood flow (CBF), but its ability to reflect CBF changes has not been extensively proved. Based on the hypothesis that alterations in CBF during apnea should be reflected in REG signals under the form of increased complexity, several entropy metrics were assessed for REG analysis during apnea and resting periods in 16 healthy subjects: approximate entropy (ApEn), sample entropy (SampEn), fuzzy entropy (FuzzyEn), corrected conditional entropy (CCE) and Shannon entropy (SE). To compute these entropy metrics, a set of parameters must be defined a priori, such as, for example, the embedding dimension m, and the tolerance threshold r. A thorough analysis of the effects of parameter selection in the entropy metrics was performed, looking for the values optimizing differences between apnea and baseline signals. All entropy metrics, except SE, provided higher values for apnea periods (*p*-values < 0.025). FuzzyEn outperformed all other metrics, providing the lowest *p*-value (*p* = 0.0001), allowing to conclude that REG signals during apnea have higher complexity than in resting periods. Those findings suggest that REG signals reflect CBF changes provoked by apneas, even though further studies are needed to confirm this hypothesis.

## 1. Introduction

The brain represents only up to 2% of the body weight in humans, while it receives up to 20% of the total cardiac output [1]. This suggests that the brain has large metabolic needs and, as it is an organ that has no mechanism to store nutrients, oxygen or water, it needs to receive a large and uninterrupted blood supply. An inadequate or drastic reduction in cerebral blood flow (CBF) would provoke brain ischemia, which is often the cause of death in traumatic head-injured patients. Furthermore, secondary brain insults are frequent in traumatic patients that could be anticipated with CBF monitoring [2].

Moreover, cerebral ischemia and neuronal damage are two critical adverse events during anesthesia. In this way, even though encephalic vascular accidents are infrequent during common surgeries, complex procedures present a higher risk [3]. The occurrence of neurologic complications during cardiac surgeries has been estimated as 2%–6% [4], often during the intraoperative period [5]. It is a relatively low occurrence but given the large number of patients undergoing this kind of procedures every year, the population at risk is considerably high.

Therefore, CBF monitoring is mandatory for critical patients and would increase safety in clinical procedures provoking alterations in CBF. Several techniques are available for that purpose; however, they are not always suitable for standard bedside monitoring either because they are invasive or extremely cumbersome and expensive. Rheoencephalography (REG) is a very simple, non-invasive and inexpensive technique that allows cerebral blood flow monitoring by sending an electric current through the scalp and measuring the impedance generated by the tissues. Since empty vessels present lower electrical conductivity than vessels filled with blood, monitoring the impedance through the scalp would reflect the amount of blood flow reaching the brain. 

Several studies have been performed evaluating the ability of REG to reflect CBF changes [6], involving assessments of cerebrovascular resistance [7] and cerebral autoregulation in both animals and humans [8,9,10]. Even though REG shows consistency among subjects [11] and correlation of REG and CBF [12] has been published, its lack of absolute values and specificity during its clinical use reduced the popularity of REG [13].

One of the most relevant problems that arise from REG signals is the interferences provoked by movements, respiration and contamination with extracranial blood flow [14,15,16]. Consequently, REG recordings suitable for processing are often short and noisy, and statistics adapted to those conditions are needed to be able to extract relevant clinical information from REG. Based on the hypothesis that apnea would provoke changes in REG signals under the form of increased complexity, several entropy metrics robust in noisy environments are assessed for REG analysis during breath holding. 

Pincus et al. [17] presented approximate entropy (ApEn), as a relative entropy metric suitable for short and noisy datasets, applicable to biomedical signals. ApEn approximates the exact regularity statistic Kolmogorov–Sinai entropy and reflects the predictability of a time series by exploring repetitive patterns in the data. It has been extensively used in heart rate variability (HRV) analysis as for example to detect heart failure [18] or to identify differences in HRV in diabetic patients [19]. Moreover, ApEn has also been used to study the electroencephalograph (EEG) regularity during sleep [20,21] and under sevoflurane anesthesia [22]. Even though those clinical applications have shown the ability of ApEn to correlate with physiological conditions, there is lots of controversy about its use. It has been reported to be inconsistent, lower than expected for short records and thus dependent on the length of the time series [23]. Furthermore, to compute the ApEn value of a time series, three parameters need to be defined: the segment length (N), the embedded dimension (m) and the noise threshold (r). The choice of those three parameters influence the ApEn result, therefore limiting its use to relative measurements. 

In order to compensate for the limitations of ApEn, Richman and Moorman [23] proposed a new entropy metric, called SampEn. The main difference of the computation of ApEn and SampEn is that SampEn does not count self matches. However, it still requires the a priori definition of the same parameters N, m and r.

SampEn has been used in different type of biomedical signals, as for example to characterize human gait signals [24], as a detector of driving fatigue in HRV signals [25] or to study EEG brain maturation in newborns [26]. Advantages of SampEn over the use of ApEn have also been reported, indicating that ApEn presents inconsistencies that are avoided by using SampEn instead [27] and that it is a better choice for short datasets [28]. 

Both ApEn and SampEn rely on the Heaviside function to define the similarity between two patterns. Due to its binary output, pairs of patterns are either included or rejected before the entropy calculation. In contrast, FuzzyEn was defined as a new entropy metric [29], in which the Heaviside function classifying the patterns as similar or not is replaced by a fuzzy function that computes a membership coefficient ranging from 0 to 1, where 1 maximizes the membership likelihood. Consequently, in addition to the selection of N, m and r, FuzzyEn requires a fourth parameter, n, which is the gradient of the boundary of the exponential function used to assess similarity. When compared to ApEn and SampEn, FuzzyEn outperformed the other measures in electromyogram (EMG) signals characterization [29], as well as in Alzheimer’s disease detection in electrocardiographic (ECG) signals [30].

Those three Entropy metrics rely on the selection of several parameters and there is lots of controversy around how they should be selected and the bias they introduce in the final entropy values. Even though some methods have been proposed to determine those values [31,32,33,34], no consensus has been reached so far. For that reason, in this paper, other metrics will be used not requiring the definition of so many parameters: Shannon Entropy (SE) and corrected conditional entropy (CCE). 

SE was introduced by Shannon to be applied in the information theory domain [35] and reflects the regularity of the information generated by a defined source. For its use in biomedical applications, the parameters to be defined are the signal length to be considered and the number of quantization levels used for signal discretization. Additionally, in some cases, SE is applied to short sequences of symbols rather than at a sample level and requires therefore the dimension of the data segments to be analyzed. SE has provided successful results when applied to EEG signals for person identification [36] and monitoring of intrapartum fetal heart rate dynamics [37]. 

CCE is an entropy measure introduced by Porta [38] that reduces the bias of regularity existing in conditional entropy. It is based on the definition of SE and has been used mainly on HRV signals, in some cases showing the expected trends but without statistical significance [39,40], and in others providing successful results, such as the ones obtained by Viola et al. [41], describing a reduction in complexity of HRV signals during Rapid Eye Movement (REM) sleep with aging. 

The entropy measures herein presented have not been previously applied to REG signals to the extent of the knowledge of the authors, but they have been extensively used for diagnosis purposes in other biomedical signals, such as the previously mentioned examples, mainly on EMG and HRV. Nonetheless, entropy measures have been applied to the study of plethysmography signals, which also reflect a pulse wave and are therefore closer to REG signals than EMG and HRV. For instance, Pham et al. [42] proved that SampEn of plethysmography records is a good predictor of mental disorder detection, therefore proving the usefulness of entropy assessment in pulse waves.

The main goal of this work is to study if entropy metrics applied to REG signals can detect changes in CBF during breath holding -apnea- and analyze which parameters would optimize the results. The underlying hypothesis is that entropy would increase during apneas, since under those circumstances CBF changes take place, altering the regular baseline pattern of REG signals and thus reducing regularity and increasing entropy. 

## 2. Materials and Methods 

### 2.1. Entropy Definitions

This section provides information on the algorithms used for entropy calculations. Different entropy metrics will be calculated and tested: ApEn, SampEn, FuzzyEn, SE and CCE. The parameters involved in the entropy evaluation will be a priori identified: the embedding dimension (m), the signal length (N), the multiplicand of the standard deviation to define the noise level (r), the gradient of the fuzzy membership function (n) and the number of quantization levels (ε).

#### 2.1.1. Shannon Entropy

The Shannon entropy (SE) [35] assesses the amount of information generated by a system. It can be used either locally or globally [43] and, for consistency with the other entropy metrics evaluated in this work, SE will be applied to consecutive patterns of length m. Hence, from a time series *x*(*n*) of length N, quantized in ε levels, a phase space reconstruction with dimension m is built, resulting in a set of vectors $x_{m}^{\varepsilon }\left(i\right)=\left(x^{\varepsilon }\left(i\right),x^{\varepsilon }\left(i-1\right),\ldots ,x^{\varepsilon }\left(i-m+1\right)\right)$. The SE of the time series is then computed as

(1) $$SE\left(m,\varepsilon \right)=-\sum_{x_{m}^{\varepsilon }}p\left(x_{m}^{\varepsilon }\right)log\;p\left(x_{m}^{\varepsilon }\right)$$

where $p\left(x_{m}^{\varepsilon }\right)$ corresponds to the joint probability of the $x_{m}^{\varepsilon }$ pattern and the sum is performed across all the different patterns. This entropy metric requires the definition of the number of quantization levels (ε), the embedding dimension (m) and the length of the input signal (N). Thus, in this work, SE will be computed for a set of quantization levels ε ranging from 10 to 50, in steps of 10, with dimensions m from 2 to 4 and a set of signal lengths N = {1000, 2000, 3000, 4000} samples. 

#### 2.1.2. Corrected Conditional Entropy

Corrected conditional entropy [38] is based on the correction applied to the conditional entropy (CE) definition. CE is calculated as the variation of SE in two consecutive values for the embedding dimension, m:

(2) $$CE\;\left(m,\varepsilon \right)=-\sum_{x_{m-1}^{\varepsilon }}p\left(x_{m-1}^{\varepsilon }\right)\sum_{x_{m}^{\varepsilon }}p\left(x_{m}^{\varepsilon }/x_{m-1}^{\varepsilon }\right)log\;p\left(x_{m}^{\varepsilon }/x_{m-1}^{\varepsilon }\right)$$

where the first term sums across all the different patterns $x_{m-1}^{\varepsilon }$ and $p\left(x_{m-1}^{\varepsilon }\right)$ corresponds to the joint probability of the $x_{m-1}^{\varepsilon }$ pattern and the second term covers all m samples in the pattern, with $p\left(x_{i}^{\varepsilon }/x_{m-1}^{\varepsilon }\right)$ representing the joint probability of the m-th pattern conditioned to the preceding m-1 patterns. Therefore, CE (3) can be formulated as a function of SE:

(3) $$CE\left(m,\varepsilon \right)=SE\left(m,\varepsilon \right)-SE\left(m-1,\varepsilon \right)$$

Porta et al. proposed in [38] a correction to CE in order to compensate for unique patterns that should theoretically increase entropy but reduce it when using the CE definition. The proposed correction consists in adding a corrective term and defining CCE as:

(4) $$CCE\left(m,\varepsilon \right)=CE\left(m,\varepsilon \right)+SE\left(1,\varepsilon \right)\;perc\left(m,\varepsilon \right)$$

where $perc\left(m,\varepsilon \right)$ is the percentage of single points in the m-dimensional space. Moreover, the same authors propose the use of the minimum of the CCE entropy, CCEmin, as an approximation to the entropy of the signal, avoiding having to define the value for m in advance for the entropy calculation [44]. Additionally, they introduced the regularity index *ρ*, computed as:

(5) $$\rho =1-\min\left(NCCE\left(m,\varepsilon \right)\right)$$

To estimate the overall regularity of a time series. In equation (5), NCCE refers to the normalized CCE by SE(1,ε), resulting in a regularity index providing values between 0 and 1, representing maximum and minimum complexity, respectively. 

CCE and the regularity index *ρ* were computed for all the signals in the experimental dataset. Analogous to the parameter set chosen for SE, CCE was computed with embedding dimension m from 2 to 4, quantization levels ε of 10, 20, 30, 40 and 50 while the length N of the segments used ranged from 1000 to 4000, in steps of 1000 samples. 

#### 2.1.3. Approximate Entropy

ApEn [17] allows us to quantify the regularity of a time series without the need of previous knowledge of the dynamics of the system [43], resulting in larger values for increasing complexity in the data. ApEn reflects the likelihood that patterns that are close, within a defined distance r, in a m-dimensional space remain close within the same tolerance when defined in a m+1 dimensional space. 

Given a digital signal u(n), with length of N samples, values for the embedded dimension m and the filtering level r are fixed a priori. A set of vectors, x, in the R^*m*^ dimensional space are then created:

(6) $$x_{i}=\left[\begin{array}{ccc} u\left(i\right), & \cdots , & u\left(i+m-1\right) \end{array}\right]$$

For each i, 1 ≤ i ≤ N-m+1, an estimation of the correlation integral C_i_^m^(r) is computed as:

(7) $$C_{i}^{m}\left(r\right)=\frac{number\;of\;j\;such\;that\;d\left[x\left(i\right),x\left(j\right)\right]\leq r}{N-m+1}$$

where the distance between x(i) and x(j) is defined as:

(8) $$d\left[x^{m}\left(i\right),x^{m}\left(j\right)\right]=\underset{k=1,2,\ldots ,m}{\max}\left(\left|u\left(t_{i+k-1}\right)-u\left(t_{j+k-1}\right)\right|\right)$$

Finally, ApEn is calculated as:

(9) $$ApEn\left(m,r,N\right)={ɸ}^{m}\left(r\right)-{ɸ}^{m+1}\left(r\right)$$

where

(10) $${ɸ}^{m}\left(r\right)={\left(N-m+1\right)}^{-1}\;\sum_{i=1}^{N-m+1}log\;C_{i}^{m}\left(r\right)$$

The performance of ApEn depends on the choice for the input parameters r and m, as well as the length of the time series to be compared. Since noise smaller than r is filtered out, ideally r should be small enough to preserve the information of the dynamics of the system, but very small values would compromise the calculation of conditional probabilities [45]. Regarding the choice for m, larger values are preferred but it shall be considered that its selection is limited by the length of the time series (N), since N should be between 10^*m*^ and 30^*m*^ points [46,47].

ApEn values can vary significantly for r and m values, therefore it shall be used for systems comparison. Typical values for m are m = 2 and m = 3, while selected values for r depend on the type of signals to which this technique is applied [17]. The most commonly used combination is m = 2 and r = 0.2 (20% of the standard deviation) [43]. Pincus et al. [17] obtained significant results on the comparison of HRV signals of healthy and sick infants using r values ranging from 0.1 to 0.25 while Chen at al. used r = 0.3 to successfully distinguish EMG signals originated by four different movements [29]. 

Even though several algorithms have been published to overcome the difficulties in the choice of r [33,34], when comparing ApEn values for two or more groups the optimal r value could be different in each group and lead to inconsistent results [48]. Therefore, experimental analysis is recommended to identify the best r for each application. 

It should also be taken into account that ApEn is a biased statistic, strongly dependent on the signal length and lacking of consistency [23], providing unexpected ApEn variations for different pairs of m and r values [47]. The bias is due to the concavity of the logarithmic function, as well as to the fact that ApEn counts self matches when computing the correlation integral [45]. 

For the analysis of REG signals in apnea and baseline recordings, considering that data sequences available were 4000 samples length, N values of the analyzed time series ranged from 1000 to 4000, in steps of 1000. For the parameter m, it was limited to m = 2, m = 3 and m = 4, the last one exceeding the N ≤ 10^*m*^ criteria. Finally, chosen r values covered the range of 0.05 to 0.3 times the standard deviation of the input signal. 

#### 2.1.4. Sample Entropy

The entropy metric SampEn [23] intends to surpass the constraints presented in ApEn by excluding self matches in the entropy calculation and, therefore, reducing computation times. The algorithm follows the same initial steps presented for ApEn, but when computing the correlation integral self-matches are excluded, as shown in Equation (11).

(11) $$C_{i}^{m}\left(r\right)=\frac{number\;of\;j\;such\;that\;d\left[x\left(i\right),x\left(j\right)\right]\leq r\;and\;i\neq j}{N-m+1}$$

Lastly, ${ɸ}^{m}\left(r\right)$ is defined as

(12) $${ɸ}^{m}\left(r\right)={\left(N-m\right)}^{-1}\;\sum_{i=1}^{N-m}logC_{i}^{m}\left(r\right)$$

and SampEn is calculated as the difference between the logarithms of ${ɸ}^{m}\left(r\right)$ and ${ɸ}^{m+1}\left(r\right):$

(13) $$SampEn\left(m,r,N\right)=\log{ɸ}^{m}\left(r\right)-\log{ɸ}^{m+1}\left(r\right)$$

SampEn requires the a priori definition of the same parameters listed for ApEn - N, m and r – and those are typically coincident with the ones used for ApEn (i.e., m = 2, r = 0.2). However, even though some authors consider the same criteria can be used for both SampEn and ApEn [49], other publications suggest that they should be explored independently since algorithms proposed for the choice of r in ApEn are not applicable for SampEn [50]. Moreover, appropriate values for m and r depend of the type of signal under analysis [49].

For instance, Lake et al. [51] studied the selection of m and r parameters for neonatal HRV analysis, concluding that the best pair of values was m = 3 and r = 0.2. In contrast, while applying the SampEn algorithm to characterize the effects of exercise in RR and QT intervals, Lewis et al. [52] explored different combinations of r and m values to finally chose m = 2 and r between 0.1 and 0.15. Higher r values have also been considered as optimal, as for example in the atrial fibrillation organization analysis presented by Alcaraz et al. [49], in which after identifying several combinations providing good classification results, the best values were considered to be m = 3 and r between 0.3 and 0.4.

SampEn overcomes the bias problem detected in ApEn as well as its inconsistencies, such that if SampEn of one signal (x_1_) is higher than the value obtained with another signal (x_2_) for a pair of m and r values, a new m-r pair would still provide higher SampEn values for the signal x_1_ [51]. Nonetheless, Castiglioni et al. [50] detected inconsistencies in SampEn calculations when studying mechanomyographic signals for certain m values, and Yentes et al. [28] published similar findings for some r choices, suggesting that under certain conditions SampEn can also be affected by inconsistencies.

Controversy around adequate m and r values and the existence of inconsistencies in SampEn calculations, requires that for a new type of signals, such as REG signals, an analysis of the effect of m, r and N is performed. Therefore, in this work, the same values suggested for ApEn will be used to explore the ability of SampEn to detect apnea periods in REG signals: a range of m (from 2 to 4), r (from 0.1 to 0.3) and N (from 1000 to 4000). 

#### 2.1.5. Fuzzy Entropy

ApEn and SampEn share a definition of similarity in which data segments with distances lower than the threshold value r are considered as positive matches, while others are rejected and not considered for the calculation. Even though ApEn includes self-matches and SampEn does not, in both cases a Heaviside function is used to assess similarity. In contrast, the definition of FuzzyEn [29] relies on a degree of similarity between 0 and 1. This similarity is based on the concept of fuzzy membership as defined by Zadeh [53] and results in a weaker influence of the choice of r in the final entropy calculations [43].

Besides the use of fuzzy membership calculations, the FuzzyEn algorithm also differs from ApEn and SampEn in the way it creates the set of m-dimensional vectors. Given a time series N-samples length u(n), vector sequences are defined as:

(14) $$x_{i}^{m}=\left\{u\left(i\right),u\left(i+1\right),\cdots ,u\left(i+m-1\right)\right\}-u0\left(i\right),$$

where $u0\left(i\right)$ represents the baseline trend and is computed as

(15) $$u0\left(i\right)=\frac{1}{m}\sum_{j=0}^{m-1}u\left(i+j\right)$$

The distance between two vectors, $x_{i}^{m}$ and $x_{j}^{m}$, is defined as the maximum distance among all the scalar components of the vector $d_{ij}^{m}$. A matrix, $D_{ij}^{m}$ is built, containing the similarity degrees for all pairs of r and n (width and gradient of the boundary of the exponential function, respectively)

(16) $$D_{i,j}^{m}=µ\left(d\left({\bar{x}}_{i}^{m},{\bar{x}}_{j}^{m}\right),n,r\right)$$

where µ is the exponential fuzzy function:

(17) $$µ\left(x,n,r\right)=e^{-{\left(\frac{x}{r}\right)}^{n}}$$

Finally, the function ɸ^*m*^ is calculated as:

(18) $${ɸ}^{m}=\frac{1}{N-m}\sum_{i=1}^{N-m}\sum_{j=1,j\neq i}^{N-m}\frac{D_{i,j}^{m}}{N-m-1}$$

and the fuzzy entropy is computed as:

(19) $$FuzzyEn\left(m,r,n,N\right)=\ln{ɸ}^{m}-\ln{ɸ}^{m+1}.$$

FuzzyEn, therefore, needs four parameters to be computed: N, m, r and n values. Typical values for N, m and r are coincident with the ones used for SampEn and ApEn, even though dependence on r is less critical due to the substitution of the Heaviside function by the fuzzy membership calculation. Regarding the values for n, only small values guarantee a good approximation of entropy [43], being n = 2 the most frequently used [29,54,55]. 

For this application on REG signals, N, m and r ranges tested were the same ones proposed for ApEn and SampEn, while n values ranged from 2 to 10.

### 2.2. Experimental Protocol

This work is based on a previously published dataset [56]. A group of 16 young healthy volunteers signed an informed consent for REG data recording during apnea and baseline periods. This study was carried out following the principles of the Declaration of Helsinki and the corresponding protocol approved by the local Institutional Review Board and Ethics Committee. Participants were aged 25.4 ± 3.6 years, 59.6 ± 6.8 kg weight and 166.9 ± 8.3 cm height, including 8 males and 8 females.

The qCO monitor (Quantium Medical, Spain) was used for cerebral impedance monitoring. Two pairs of electrodes were placed in the subject’s temples, one pair in each side, containing an electrode emitting current and a second one sensing the output signal. A 50 kHz, 1 mA current was used for excitation and the obtained REG signal was recorded at 250 samples/s. 

Subjects were asked to relax in supine position until a stable REG signal was obtained. Afterwards, data recording started, repeating twice the sequence consisting of 3 min of resting period followed by 1 min of breath holding. In case volunteers were unable to complete the 1 min apnea, they were required to raise their hand to inform the investigators and start the 3 min resting period. 

### 2.3. Data Analysis

**Figure 1:**
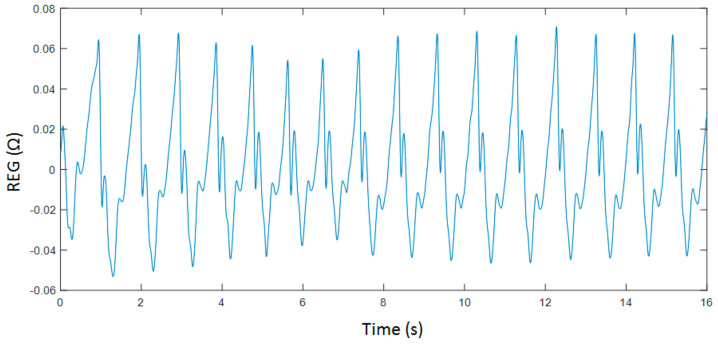
Filtered rheoencephalography (REG) signal collected during breath holding.

Even though entropy measures are known to be robust in the presence of limited amounts of noise, the recorded signals were filtered to reduce the influence of powerline interferences in the computed parameters and to filter out slow drifts provoked by respiration as well as other direct current (DC) fluctuations. Two Chebyshev type II filters were used, one of them being a 4th order high-pass filter with a stop band frequency of 0.1 Hz and the other one being an 8th order low-pass filter with stop band frequency at 20 Hz. Moreover, filtered signals were screened and detected artefacts were rejected to finally select data segments of 4000 samples. An example of a pre-processed REG recoding is shown in Figure 1.

Finally, 53 sequences were selected, 29 belonging to apnea recordings and 24 from baseline periods. The average main frequency of the recorded signals was 1.10 ± 0.47 Hz (mean ± standard deviation), resulting in a cardiac cycle of 245 ± 57 samples. The dynamic range of the recorded REG waves was 0.089 ± 0.028 Ω (95% confidence interval). No differences were observed between groups in terms of amplitudes or heart cycle duration. 

All entropy metrics were computed for each input parameter combination indicated in Table 1, and their ability to distinguish baseline and apnea sequences was assessed by hypothesis testing, using either Student *t*-tests or Mann–Whitney tests, for normal and non-normal distributions, respectively. Normality was determined using a Lilliefors test. The statistical significance threshold was set at *p* < 0.05, and Bonferroni corrections were applied resulting in a final threshold of *p* < 0.025. Additionally, the area under the curve (AUC) of the receiver operating characteristic (ROC) and the classification accuracy (acc) were also computed. 

REG signals processing is typically based on the analysis of the geometry of the pulse waves, by means of detecting local maximums and minimums as well as other features extracted from the time series [57,58]. In order to determine if the entropy metrics herein proposed outperform this classical analysis, the following set of features were extracted from the recordings:

- Maximum amplitude (Max)
- Minimum amplitude (Min)
- Amplitude range (Range)
- Slope of the increasing edge (α)
- Area under the curve of each cardiac cycle (Area)
- Time between two consecutive maximums (Δtmax)
- Time between two consecutive minimums (Δtmin)
- Time between a minimum and its consecutive maximum (Δtmin-max)

Moreover, the derivatives of the time series were also computed and the maximum value of the derivative in each cycle (δmax) and the range of the derivative (δrange) were computed and analyzed. The median value of those features in each recording was considered for analysis and subject to hypothesis testing under the same assumptions used for the entropy metrics. 

## 3. Results

### 3.1. Parameters Selection for Each Entropy Metric

The evolution of the entropy metrics ApEn(m,r,N), SampEn(m,r,N), FuzzyEn(m,n,r,N), SE(N,m,ε), CCE(N,m,ε) and *ρ*(N,ε) as a function of the parameters selection is herein presented, as well as their ability to differentiate between apnea and baseline signals. 

**Figure 2:**
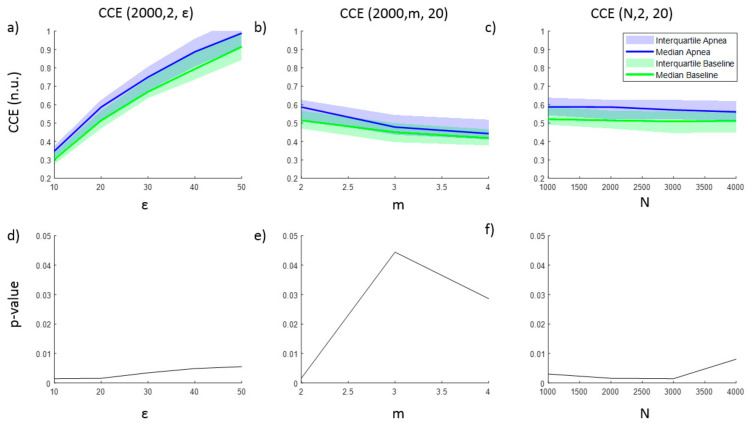
Corrected conditional entropy (CCE(N, m, ε)) values of apnea and baseline recordings as a function of (**a**) the quantification intervals (ε), (**b**) the embedding dimension (m) and (**c**) the signal length (N). The corresponding statistical significance (*p*-value) of the differences between apnea and baseline recordings is presented in (**d**), (**e**) and (**f**), respectively.

The entropy CCE and the regularity index *ρ* resulted in statistically significant results for apnea detection, while none of the parameter combinations tested for SE was able to identify apneas. Results for CCE as a function of ε, m and N are provided in Figure 2, together with the corresponding *p*-value illustrating the ability of CCE to distinguish between apnea and baseline recordings. As the number of quantization levels ε increases, CCE increases for both apnea and baseline periods (Figure 2a), but the *p*-value decreases (Figure 2d), showing a minimum for ε = 10 and ε = 20 levels. Regarding the embedding dimension m, CCE decreases as m increases, providing the best statistical significance for m = 2 ((Figure 2b,e)), while CCE remains almost stable for increasing segments length (N) (Figure 2c). Segments with lengths of 2000 and 3000 samples provide the lowest *p*-value.

**Figure 3:**
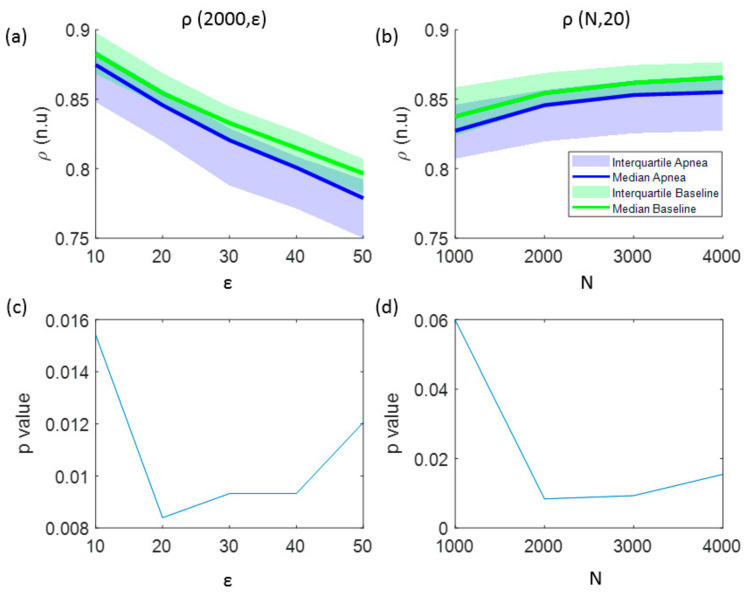
The influence of the signal length and the number of quantization levels in the regularity index (*ρ*) is analyzed in: (**a**) values of *ρ* as a function of the number of quantification intervals (ε) and (**b**) values of *ρ* as a function of the signal length (N). The results of the statistical analysis (*p*-value) comparing apnea and baseline signals using this entropy indexes are shown in: (**c**) *p*-values versus the number of quantification intervals and (**d**) *p*-values versus the signal length.

In addition to the analysis of CCE values, Figure 3 illustrates the results for the regularity index *ρ*. A monotonic decrease in regularity was observed for increasing number of quantification intervals (ε), showing higher regularity for baseline recordings (Figure 3a). The effect of increasing the signal length (N) is depicted in Figure 3b, showing an increase of regularity as N increases. Regarding the influence of the number of the quantification intervals in the statistical significance of the results, using ε ≤ 50 intervals kept *p*-value lower than the significance threshold (*p* < 0.025) for signal lengths of N = 2000 samples, as shown in Figure 3c. However, for a fixed number of quantification intervals ε = 20, the regularity index *ρ* is statistically significant for signal lengths N ≥ 2000 samples (Figure 3d). Therefore, optimal parameters for *ρ* calculation to detect apneas are ε = 20 quantification steps for signals of N = 2000 samples.

**Figure 4:**
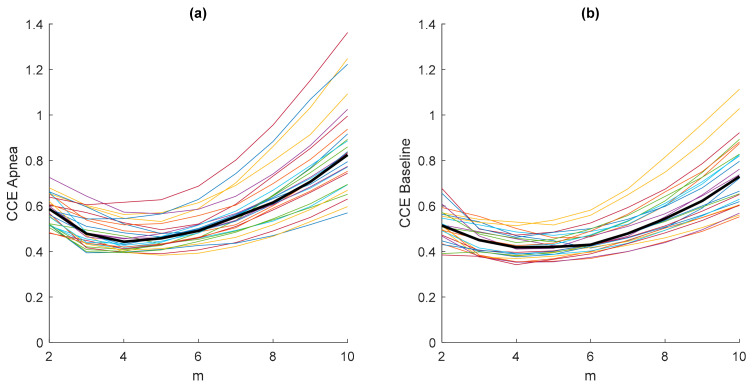
Values of the entropy CCE (ε = 20; N = 2000) as a function of the embedding dimension m for all apnea (**a**) and baseline (**b**) recordings, including their median values (thick black line).

The results in Figure 3 were obtained considering an embedded dimension m = 10, assuming that the minimum value CCEmin of the CCE would fall into this m range. To prove this assumption, a study of the entropy CCE varying the values of the embedded dimension m is presented in Figure 4. Each plotted CCE curve in Figure 4a belongs to an apnea recording and each in Figure 4b to a baseline recording. It can be observed that the minimum entropy takes place for m < 10 in both apnea and baseline signals. Furthermore, the location of the minimum CCE is not affected by the type of signal (apnea or baseline), as shown in Figure 4, and the median CCE values of the apnea signals are higher than the median values obtained from the baseline recordings.

**Figure 5:**
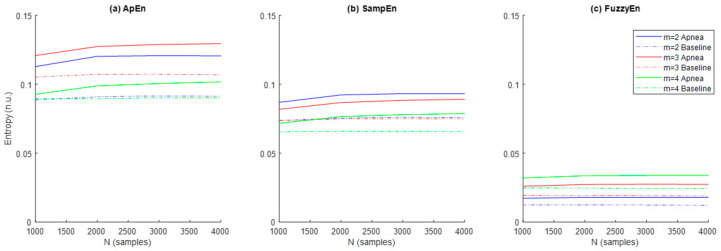
Values of the entropies (**a**) ApEn, (**b**) SampEn and (**c**) FuzzyEn as a function of the number of samples (N) and the dimension (m) for apnea (solid line) and baseline segments (dashed line).

Results referred to the study of ApEn, SampEn and FuzzyEn entropies are shown in Figure 5. In order to explore the effects of m and N, the parameter r was initially fixed to 0.3 as recommended in [29]. Entropy values were higher for apneas when compared to baseline for all entropy metrics and parameter combinations. ApEn provided the highest entropy values, followed by SampEn and FuzzyEn, respectively. Both ApEn and SampEn provided lower values for recordings of N = 1000 samples and remained approximately stable for recordings of N = 2000 samples or larger.

The ability of the three entropy metrics to distinguish between apnea and baseline segments was assessed by the *p*-value resulting from the hypothesis testing (Table 2). FuzzyEn provided statistically significant differences between both types of signals in all parameter combinations tested for m and N, while statistical significance for ApEn was limited to m = 2 and m = 3 for any sequence length and for SampEn was limited to m= 2 for a signal length of N ≥ 2000 samples. Therefore, parameter values m = 2 and N = 2000 were selected as the most appropriate across all entropy metrics for apnea detection in REG signals. 

**Figure 6:**
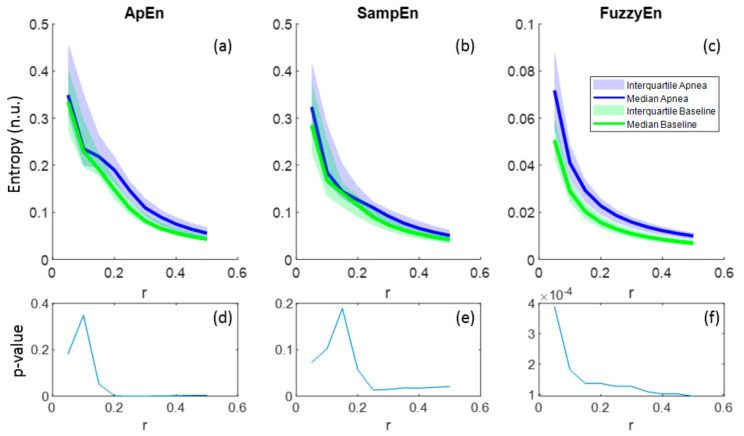
Entropy values of ApEn(2,r,2000), SampEn(2,r,2000) and FuzzyEn(2,2,r,2000) as a function of r for apnea and baseline recordings (**a**–**c**) and the corresponding *p*-values (**d**–**f**).

Regarding the parameter r, all entropies showed lower values as r increased and this behavior was common for both apnea and baseline signals (Figure 6a–c). In the case of FuzzyEn, *p*-values decreased monotonically with r, proving a better differentiation between apnea and baseline as r grows, even though FuzzyEn provided *p*-value< 0.025 for all r (Figure 6f). Instead, ApEn and SampEn needed at least r = 0.2 and r = 0.25, respectively, to provide significant results, showing both a minimum for r = 0.25 (Figure 6d–e). For that reason, r = 0.25 was considered a suitable value for apnea detection in REG signals.

**Figure 7:**
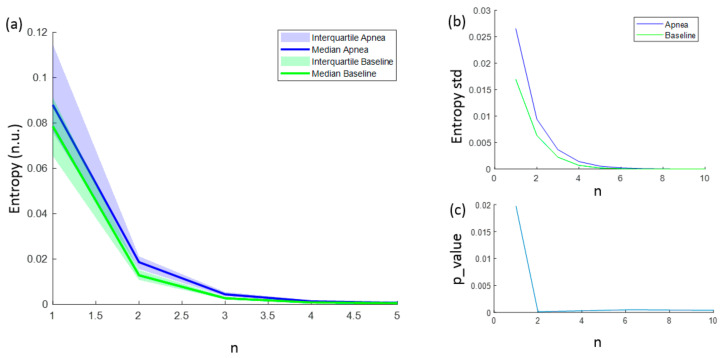
(**a**) Median FuzzyEn values as a function of n, including the 25–75 interquartile range (colored area); (**b**) standard deviation of FuzzyEn as a function of *n*; (**c**) *p*-value obtained comparing FuzzyEn values in apnea and baseline groups as a function of n.

The entropies ApEn and SampEn are fully characterized with values for N, r and m. However, for FuzzyEn, a fourth parameter (n) needs to be considered. FuzzyEn showed decreasing values for increasing n values, as shown in Figure 7a, and the standard deviation of computed entropies only tended to 0 for values of n higher than 6 (Figure 7b). In order to select the best n value for apnea detection, the statistically significant level was calculated comparing the FuzzyEn values of apnea from baseline group. FuzzyEn had the minimum *p*-value at n = 2 and hence this was considered the best choice (Figure 7c).

**Figure 8:**
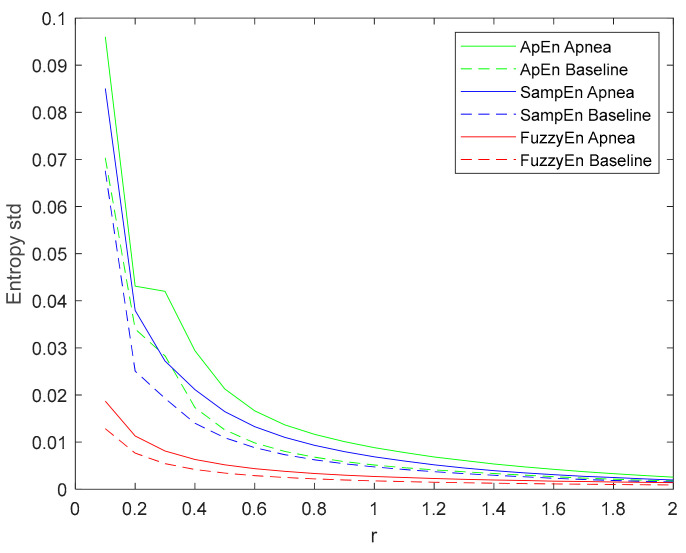
Standard deviation of ApEn(2, r, 2000), SampEn(2, r, 2000) and FuzzyEn(2, 2, r, 2000) as a function of r comparing baseline and apnea segments.

The standard deviation of the entropy metrics provides an assessment of their stability. Moreover, its evolution of r is used to determine their consistency [29]. Therefore, the evolution of the standard deviation of the three entropy metrics (ApEn, SampEn and FuzzyEn) as a function of the parameter r is depicted in Figure 8. All of them decrease with increasing r, showing a higher standard deviation value for apneas than baselines. FuzzyEn showed the lowest standard deviation, followed by SampEn. It is worth noting that both FuzzyEn and SampEn decreased monotonically while ApEn showed an almost flat behavior for r values around 0.3 in the apnea signals. This phenomenon was less pronounced in baseline recordings, but an inflection point can be observed in the same r range.

### 3.2. Final Parameter and Entropy Values

Results for all tested entropy metrics are included in Table 3. The values of the parameters that best describe these entropies when comparing apnea and baseline recordings are included. All these entropy metrics show increased values for apnea recordings, indicating an increased signal complexity. It should be noted that the index *ρ* presents the opposite behavior, since it reflects regularity instead of complexity.

**Figure 9:**
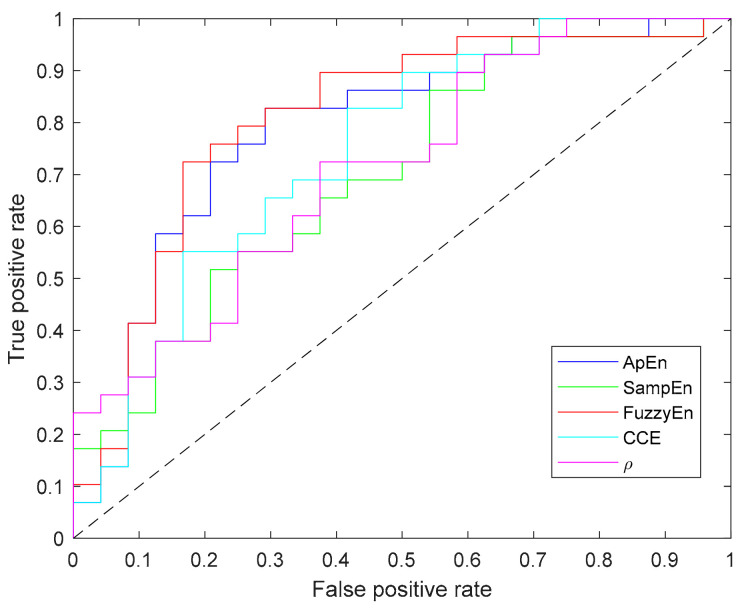
Receiver operating characteristic (ROC) curves of all entropy metrics providing statistically significant differences between apnea and baseline recordings.

Since Shannon entropy did not provide significant results for any parameter (N, m, ε) combination it has not been included in this table. In addition to the *p*-value computed for each metric, Table 3 contains the values of area under the curve (AUC) and accuracy (acc), in which FuzzyEn outperforms other entropy metrics. Moreover, Figure 9 depicts the ROC curves for all the entropy metrics summarized in the table. 

**Figure 10:**
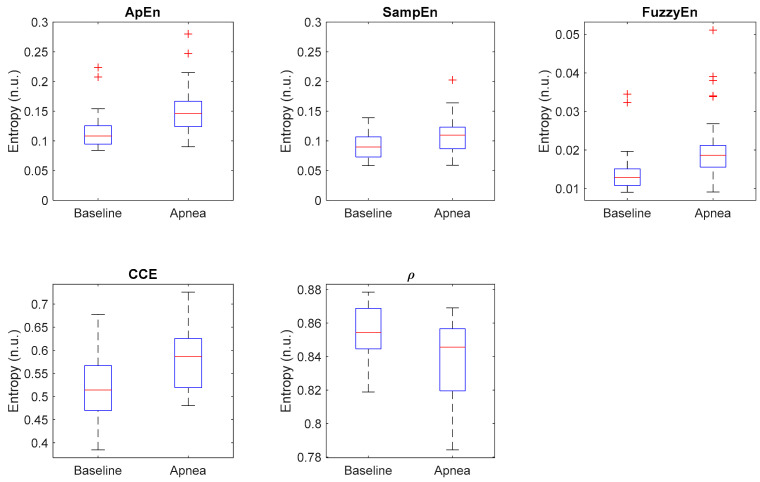
Boxplot of all selected entropy metrics, showing the median values (horizontal red lines) and outliers (red crosses).

Additionally, Figure 10 shows the distribution of each entropy metric for baseline and apnea groups. CCE and *ρ* present the highest dispersion of values, while Apen, SampEn and FuzzyEn have less dispersed distributions but with many outliers, specially Apen and FuzzyEn. Those results suggest that even though the selected metrics provide statistically significant differences in apnea and baseline recordings, individual differences should be noted.

Finally, results obtained applying the classical REG analysis based on geometric features extraction are provided in Table 4. None of the proposed features showed statistically significant differences between apnea and baseline signals, suggesting that entropy metrics outperform the classical analysis of REG waves for apnea detection. 

## 4. Discussion

All entropy metrics proposed, except for SE, provided evidence regarding the increased irregularity in apnea signals when compared to baseline recordings. However, those results were shown to be dependent on the choice of the parameters needed for each entropy metric calculation. For instance, CCE values increased with an increasing number of quantization intervals, and decreased with increasing m, while remained stable with increasing sequence length. The regularity index (*ρ*) decreased with the number of quantization levels, in accordance with the evolution of CCE since *ρ* reflects regularity instead of entropy. However, *ρ* increased with increasing signal length indicating that a fewer number of new patterns were detected when signal length was extended. Those results are consistent with those published by Porta et al. [38], since REG waves show a quasi-periodic pattern. However, it should be noted that when using REG signals, the optimal number of quantization levels providing a better differentiation between apnea and baseline recordings, ε = 20, is higher than the one proposed by Porta in his work, ε = 6.

Considering the performances of CCE and the regularity index *ρ*, the latter provided the lowest *p*-value when tested for differences between apnea and baseline recordings. This allows us to conclude that the normalization step in the definition of *ρ* enhances comparative results. 

Even though SE and CCE are both derived from the original definition of the Shannon entropy, CCE provides significant results while SE does not. This different performance of SE and CCE exists because SE reflects the distribution of the patterns in a given sequence while CCE assesses differences between consecutive patterns. This phenomenon has been analyzed previously in other publications [59], referring to SE as an entropy measure and conditional entropy as an entropy rate. 

Regarding the results for ApEn, SampEn and FuzzyEn, they all decrease with increasing r threshold, but their behavior with increasing time series length and embedding dimension differs. Figure 5a shows increasing ApEn values for longer signals in apneas while the effects of signal length in baseline recordings are negligible. The same trend can be observed for SampEn in Figure 5b, while FuzzyEn (Figure 5c) shows stable entropy values for all signal lengths. SampEn was reported to be independent of signal length while ApEn is known to provide lower entropy estimates for short recordings [23]. Considering that the effect of signal length is only present in apneas, results could be interpreted as an increasing complexity in REG signals proportional to apnea duration, rather than just a weakness of the entropy estimators. 

One of the main differences between ApEn, SampEn and FuzzyEn is their evolution as a function of the embedding dimension m. SampEn (Figure 5b) provides lower entropies for increasing m, while FuzzyEn (Figure 5c) shows the opposite behavior and ApEn does not show a consistent behavior, since the highest entropy is obtained for m = 3, followed by m = 2 and m = 4 (Figure 5a). This inconsistency in ApEn might be due to the bias inherent in this estimation. Moreover, the use of the Heaviside function might be influencing the results in such a way that softening the similarity boundary with fuzzy membership functions provides the most consistent results in terms of entropy rates as a function of the embedding dimension for a fixed r value. 

No other inconsistencies were detected in ApEn, SampEn or FuzzyEn. Some authors have reported a flip-flop effect in entropy estimations [60,61]. They observed that, given two groups of signals to be compared, some r values resulted in higher entropy for signals in one group, while other r selections provided the opposite results. No flip-flop episodes were detected in this apnea-baseline dataset. Moreover, considering the definition of Aktaruzzaman [62] of practical consistency, one can conclude that the three metrics were consistent since always identified higher entropies in the apnea group for a broad range of input parameters. However, looking at the evolution of the standard deviation of each entropy (Figure 8), FuzzyEn provides the lowest values, followed by SampEn. For ApEn, the Entropy standard deviation is not decreasing monotonically, since it shows a plateau around r = 0.3. This suggests a higher variability of ApEn calculations when compared to the other estimators. 

ApEn provides the highest entropy values and FuzzyEn the lowest, but all of them provide significantly different results for apnea and baseline recordings, for one or more set of N, m, r and n parameters. Optimal values for apnea detection were common to ApEn, SampEn and FuzzyEn—using n = 2 for fuzzy membership functions- even though FuzzyEn showed to be less sensitive to parameter selection, providing significant results for all the parameter combinations tested. 

Recommended values for r, m and N are slightly different from those reported by other authors with different types of signals. The embedding dimension, m = 2, is coincident with most of the analysis published, but different from the one provided for plethysmograms [42], m = 7. However, due to the limited length of the recording, using embedding dimensions higher than 3 or 4 would require the use of very large r values, loosing information of the patterns in the time series. Values for the r threshold optimizing apnea detection are higher than the ones reported in other applications, usually ranging from 0.15 to 0.2 [51,52]. Regarding the value of n in the FuzzyEn algorithm, recommendations of using the smallest possible value are consistent with the results herein presented, where n = 2 provided the better statistical significance for apnea detection. 

FuzzyEn provided the best statistical significance and AUC for apnea detection in REG signals, followed by ApEn, CCE and SampEn, all of them identifying higher complexity in apnea when compared to baseline signals (as can be seen in Table 3). Previous publications have also compared the performance of different entropy metrics. For instance, Chen et al. [29] compared ApEn, SampEn and FuzzyEn in their ability to characterize surface EMG signals, where FuzzyEn outperformed the other metrics, both in terms of classification and by providing a lower standard deviation of the entropy metrics, as it is also observed in the present study. Xie et al. [54] also compared the same three entropy definitions with the objective of detecting muscular fatigue in EMG signals. FuzzyEn provided the best results while ApEn failed to detect muscular fatigue. Furthermore, while analyzing EEG in patients with Alzheimer’s disease compared to healthy subjects, FuzzyEn was also the best predictor when compared to ApEn and SampEn [30], and ApEn was again considered the poorest estimator. Even though SampEn is known to outperform ApEn [23], in this study ApEn provided a better discrimination between apnea and baseline signals. Analogously, Cuesta-Frau et al. [63] reached the same conclusion when studying body temperature records of critical patients as a predictor of survival.

All time series processed in this study were sampled as 250 Hz. It is well known that sampling frequency affects the selection of the optimal parameters for entropy calculation [49], as well as signal to noise ratio [28]. However, due to the artefacts present in the recording because of movements, reducing the sampling frequency would have limited the length of the time series and, therefore, the range of dimensions m tested for each entropy definition. Therefore, sampling frequency was not included as an input variable in the estimation of entropy in the recorded dataset. 

The use of entropy metrics applied to biosignals often aims at detecting a disease, as for example heart failure [18] or sick newborns [51] by means of the analysis of HRV signals. In those cases, lower entropies are associated to the disease condition. No previous studies on the regularity of REG signals have been published to the best knowledge of the authors. However, Pham et al. [42] analyzed plethysmograms, which share many properties with REG signals, and used the information for diagnosis purposes, aiming at detecting mental disorders. In our study, participants were healthy volunteers performing a simple respiratory challenge to provoke CBF changes. Therefore, rather than detecting a disease, entropy metrics were used to detect alterations in CBF reflected in REG waves. The results suggest that during apneas, in order to preserve a fixed amount of oxygen supplied to the brain, compensation mechanisms are activated that modify the REG pulse waves adding complexity to the signal. During baseline, oxygen and blood supply to the brain do not suffer alterations and REG signals are, therefore, more regular. 

Further studies are needed to confirm those findings, but results suggest that entropy analysis is suitable for CBF changes detection in REG signals. Moreover, this analysis outperforms the classical approach used for REG signals, based on geometric features detection in the pulse waves, that was proved to fail in detecting apneas. 

## 5. Conclusions

The findings presented in this study suggest that FuzzyEn is the entropy metric providing the best ability to distinguish between apnea and baseline in REG signals among the set of entropy metrics proposed, followed by ApEn and CCE. Nonetheless, a careful selection of the input parameters needed to compute those entropy metrics should be performed in advance, since values recommended for other applications are not suitable for REG signals. 

Moreover, entropy analysis has been shown to be more adequate for apnea detection than classical methods applied to REG signals. Even though a larger dataset and other mechanisms to alter CBF are needed to confirm those findings, REG signals seem to be carrying CBF information that can be assessed by means of complexity analysis.

## Figures and Tables

**Table 1:** Parameter combination used to calculate each entropy metric.

	Signal Length (N) (Samples)	Embedding Dimension (m)	Filtering Level (r)	Quantization Intervals (ε)	Fuzzy Function Gradient (n)
**Shannon Entropy**	1000 to 4000	2 to 4	-	10 to 50	-
**Corrected Conditional Entropy**	1000 to 4000	2 to 4 *	-	10 to 50	-
**Approximate Entropy**	1000 to 4000	2 to 4	0.05 to 0.3	-	-
**Sample Entropy**	1000 to 4000	2 to 4	0.05 to 0.3	-	-
**Fuzzy entropy**	1000 to 4000	2 to 4	0.05 to 0.3	-	2 to 10

* Only used in CCE calculation, not applicable for *ρ*.

**Table 2:** Statistical significance values of the entropies ApEn, SampEn and FuzzyEn for apnea detection as a function of the embedding dimension (m) and the signal length (N).

	N = 1000	N = 2000	N = 3000	N = 4000
***ApEn***				
m = 2	0.0044	0.0006	0.0006	0.0004
m = 3	0.0131	0.0014	0.0013	0.0004
m = 4	0.6379	0.4915	0.5376	0.3391
***SampEn***				
m = 2	0.048	0.014	0.017	0.017
m = 3	0.166	0.195	0.145	0.136
m = 4	0.387	0.280	0.183	0.172
***FuzzyEn***				
m = 2	0.00076	0.00013	0.00014	0.00012
m = 3	0.00086	0.00018	0.00016	0.00014
m = 4	0.00329	0.00042	0.00022	0.00021

**Table 3:** Mean values and standard deviation (std) of all entropy metrics when comparing apnea and baseline recordings. The values of the set of parameters that best describe these entropies are included. Statistics as *p*-value, area under the curve (AUC) and accuracy (acc) are provided to assess the ability of the entropy metrics to distinguish between apnea and baseline.

Entropy Measure	Parameters	Apnea Mean ± std	Baseline Mean ± std	*p*-Value	AUC	acc (%)
ApEn	r = 0.25 m = 2 N = 2000	0.155 ± 0.045	0.118 ± 0.035	0.0003	0.789	69.8
SampEn	r = 0.25 m = 2 N = 2000	0.111 ± 0.031	0.092 ± 0.022	0.0132	0.698	60.4
FuzzyEn	r = 0.25 m = 2 N = 2000 n = 2	0.021 ± 0.009	0.015 ± 0.006	0.0001	0.809	69.8
CCE	ε = 20 m = 2 N = 2000	0.581 ± 0.063	0.518 ± 0.075	0.0016	0.744	67.9
ρ	ε = 20 N = 2000	0.838 ± 0.024	0.854 ± 0.017	0.0084	0.713	62.3

**Table 4:** Mean values and standard deviation (std) of all the features extracted from the linear time series and p_value statistics illustrating their ability to distinguish between apnea and baseline signals.

Parameter	Units	Apnea Mean ± std	Baseline Mean ± std	*p*-Value
**Max**	Ω	0.041 ± 0.014	0.045 ± 0.017	0.356
**Min**	Ω	−0.051 ± 0.017	−0.054 ± 0.018	0.523
**Range**	Ω	0.092 ± 0.028	0.099 ± 0.033	0.376
**Δtmax**	samples	238.7 ± 22.1	254.9 ± 43.2	0.084
**Δtmin**	samples	242.11 ± 23.2	248.6 ± 38.8	0.455
**Δtmin-max**	samples	52.88 ± 27.36	60.56 ± 24.76	0.217
**α**	a.u.	0.002 ± 0.001	0.002 ± 0.001	0.406
**Area**	Ω.s	12.453 ± 4.766	13.471 ± 4.856	0.446
**δmax**	Ω/s	0.006 ± 0.002	0.005 ± 0.002	0.272
**δrange**	Ω/s	0.007 ± 0.002	0.007 ± 0.002	0.145
